# Supplementary material for: The interactome of intact mitochondria by cross-linking mass spectrometry provides evidence for coexisting respiratory supercomplexes
Source: Mol Cell Proteomics. 2017 Dec 8;17(2):216–32. doi: 10.1074/mcp.RA117.000470 (PMC5795388; doi:10.1074/mcp.RA117.000470)
Supplement: Supplemental Data [file supp_RA117.000470_133922_0_supp_23978_fzffwf.zip › spectra_annotation/mito_DR_spectra_annotation/305-1-8-1-11-1.pdf]

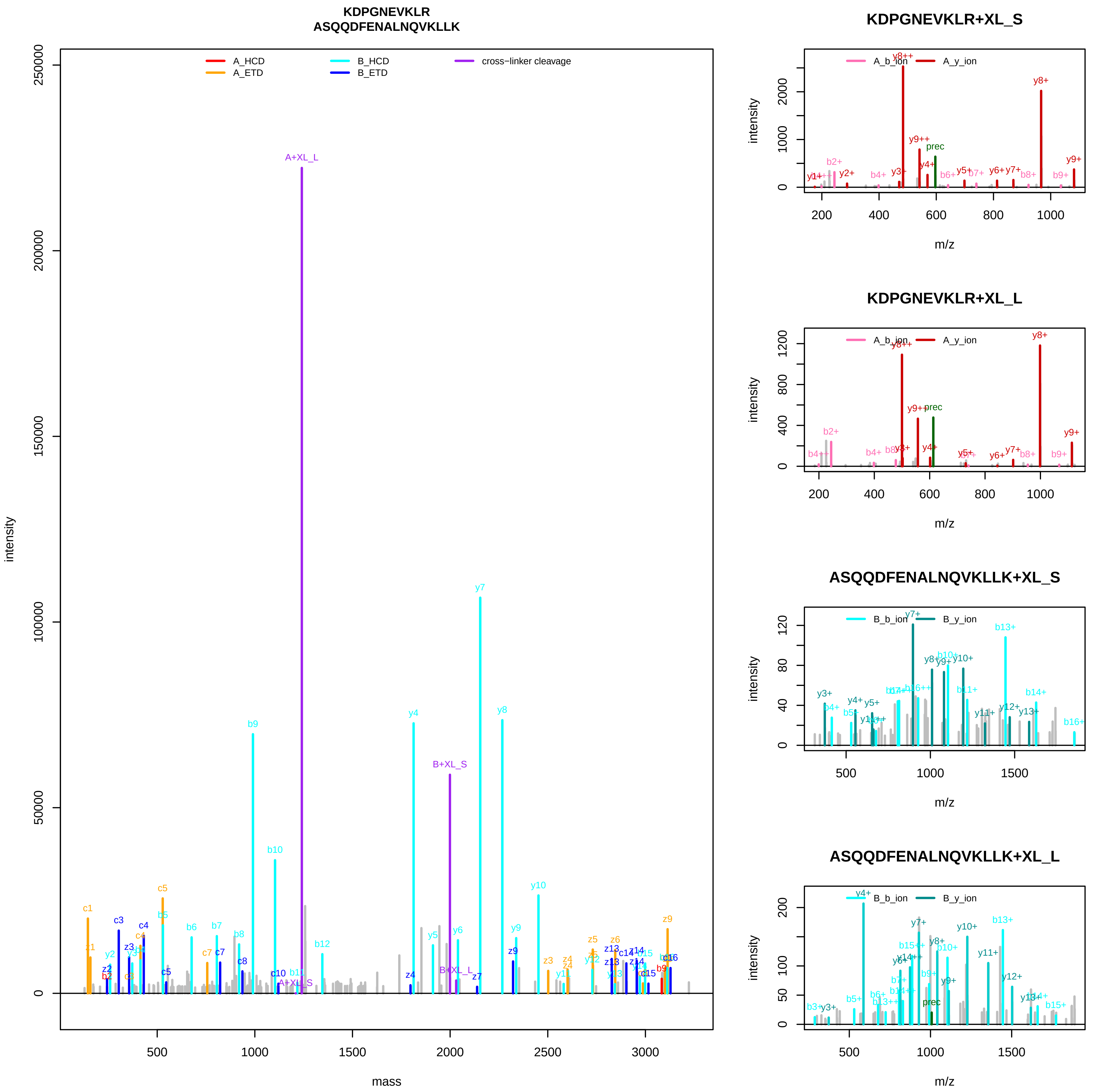

KDPGNEVKLR
ASQQDFENALNQVKLLK
A_HCD
A_ETD
B_HCD
B_ETD
cross−linker cleavage
A+XL_L
B+XL_S
A+XL_S
B+XL_L
y7
y8
y4
b9
b10
b12
y10
y9
y5
y6
z9
c1
c5
b5
b6
b7
b8
c3
c4
c7
c8
c10
z4
z7
z3
z5
z6
z13
c14
z14
b15
c15
c16
b9
z9
y12
intensity
mass
0
50000
100000
150000
200000
250000
500
1000
1500
2000
2500
3000
KDPGNEVKLR+XL_S
A_b_ion
A_y_ion
y8++
y8+
y9++
prec
y9+
b2+
y2+
y3+
y4+
b4+
b6+
y5+
b7+
y6+
y7+
b8+
b9+
y1+
KDPGNEVKLR+XL_L
ASQQDFENALNQVKLLK+XL_S
B_b_ion
B_y_ion
y7+
b13+
y8+
b10+
y9+
y10+
b11+
b14+
b16+
y3+
b4+
y4+
y5+
b5+
y11+
y12+
y13+
ASQQDFENALNQVKLLK+XL_L
y4+
y10+
b13+
y11+
y12+
y13+
b15+
b3+
b5+
prec
intensity
m/z
